# A Monotone Path Proof of an Extremal Result for Long Markov Chains

**DOI:** 10.3390/e21030276

**Published:** 2019-03-13

**Authors:** Jia Wang, Jun Chen

**Affiliations:** 1 Department of Electronic Engineering, Shanghai Jiao Tong University, Shanghai 200240, China; 2 Department of Electrical and Computer Engineering, McMaster University, Hamilton, ON L8S 4K1, Canada

**Keywords:** entropy power inequality, Karush–Kuhn–Tucker, Markov chain, mean squared error, semidefinite programming

## Abstract

We prove an extremal result for long Markov chains based on the monotone path argument, generalizing an earlier work by Courtade and Jiao.

## 1. Introduction

Shannon’s entropy power inequality (EPI) and its variants assert the optimality of the Gaussian solution to certain extremal problems. They play important roles in characterizing the information-theoretic limits of network information theory problems that involve Gaussian sources and/or channels (see, e.g., [1,2,3,4,5,6,7,8,9,10,11,12,13,14,15]). Many different approaches have been developed for proving such extremal results. Two notable ones are the doubling trick [16,17] and the monotone path argument [18,19,20,21]. Roughly speaking, the former reaches the desired conclusion by establishing the subadditivity of the relevant functional while the latter accomplishes its goal by constructing a monotone path with one end associated with an arbitrary given point in the feasible region and the other associated with the optimal solution to the Gaussian version of the problem. Though the doubling trick typically yields simpler proofs, the monotone path argument tends to be more informative. Indeed, it shows not only the existence of the Gaussian optimizer, but also the fact that every Karush–Kuhn–Tucker (KKT) point (also known as the stationary point) of the Gaussian version of the problem is in fact globally optimal. Such information is highly useful for numerical optimization.

Several years ago, inspired by the Gaussian two-terminal source coding problem, Courtade [22] conjectured the following EPI-type extremal result for long Markov chains.

**Conjecture** **1.** *Let X and Z be two independent n-dimensional zero-mean Gaussian random (column) vectors with covariance matrices $\Sigma_{X}≻0$ and $\Sigma_{Z}≻0$ respectively, and define Y=X+Z. Then, for any μ≥0,*

(1) $$\begin{array}{c} \underset{U,V:U↔X↔Y↔V}{\inf}I\left(X;U\right)-\mu I\left(Y;U\right)+I\left(Y;V|U\right)-\mu I\left(X;V|U\right) \end{array}$$

*has a Gaussian minimizer (i.e., the infimum is achieved by some (U,V) jointly Gaussian with (X,Y)). Here, U↔X↔Y↔V means U, X, Y, and V form a Markov chain in that order.*

Later, Courtade and Jiao [23] proved this conjecture using the doubling trick. It is natural to ask whether this conjecture can also be proved via the monotone path argument. We shall show in this paper that it is indeed possible. Our work also sheds some light on the connection between these two proof strategies.

In fact, we shall prove a strengthened version of Conjecture 1 with some additional constraints imposed on (U,V). For any random (column) vector S and random object ω, let $D_{S|\omega }$ denote the distortion covariance matrix incurred by the minimum mean squared error (MMSE) estimator of S from ω (i.e., $E[\left(S-E\left[S|\omega \right]\right){\left(S-E\left[S|\omega \right]\right)}^{t}]$), where ${\left(\cdot \right)}^{t}$ is the transpose operator. The main result of this paper is as follows.

**Theorem** **1.** *For any μ≥0,*

(2) infU,V:U↔X↔Y↔V(DX|Y,U,DY|X,V)∈D−μh(X|U)+μh(Y|U)+(μ−1)h(X|U,V)−h(Y|V)+h(X|V)

*has a Gaussian minimizer, where $D=\{\left(D_{a},D_{b}\right):0≺D_{a}⪯D_{1},0≺D_{b}⪯D_{2}\}$ with $D_{1}$ and $D_{2}$ satisfying $0≺D_{1}⪯D_{X|Y}$ and $0≺D_{2}⪯D_{Y|X}$.*

**Remark** **1.** *The objective functions in (1) and (2) are equivalent. Indeed,*

$$\begin{array}{c} I(X;U)-\mu I(Y;U)+I(Y;V|U)-\mu I(X;V|U)\\ \approx -h(X|U)+\mu h(Y|U)+h(Y|U)-h(Y|U,V)-\mu h(X|U)+\mu h(X|U,V)\\ =-(\mu +1)h(X|U)+(\mu +1)h(Y|U)-h(Y|U,V)+\mu h(X|U,V)+h(Y|X,U,V)-h(Y|X,V)\\ =-(\mu +1)h(X|U)+(\mu +1)h(Y|U)-I(Y;X|U,V)+\mu h(X|U,V)-h(Y|X,V)\\ =-(\mu +1)h(X|U)+(\mu +1)h(Y|U)+(\mu -1)h(X|U,V)+h(X|Y,U)-h(Y|X,V)\\ \approx -(\mu +1)h(X|U)+(\mu +1)h(Y|U)+(\mu -1)h(X|U,V)+h(X|U)-h(Y|U)-h(Y|X,V)\\ \approx -\mu h(X|U)+\mu h(Y|U)+(\mu -1)h(X|U,V)-h(Y|V)+h(X|V), \end{array}$$

*where “≈” means that the two sides are equal up to an additive constant.*

**Remark** **2.** *Conjecture 1 corresponds to the special case where $D_{1}=D_{X|Y}$ and $D_{2}=D_{Y|X}$.*

The rest of the paper is organized as follows. Section 2 is devoted to the analysis of the Gaussian version of the optimization problem in (2). The key construction underlying our monotone path argument is introduced in Section 3. The proof of Theorem 1 is presented in Section 4. We conclude the paper in Section 5.

## 2. The Gaussian Version

In this section, we consider the Gaussian version of the optimization problem in (2) defined by imposing the restriction that (U,V) and (X,Y) are jointly Gaussian. Specifically, let $U^{g}=X+N_{1}$ and $V^{g}=Y+N_{2}$, where $N_{1}$ and $N_{2}$ are two independent *n*-dimensional zero-mean Gaussian random (column) vectors with covariance matrices $\Sigma_{1}≻0$ and $\Sigma_{2}≻0$, respectively. It is assumed that $\left(N_{1},N_{2}\right)$ is independent of (X,Y); as a consequence, the Markov chain constraint $U^{g}↔X↔Y↔V^{g}$ is satisfied. Clearly,

$$\begin{array}{c} -\mu h\left(X|U^{g}\right)+\mu h\left(Y|U^{g}\right)+\left(\mu -1\right)h\left(X|U^{g},V^{g}\right)-h\left(Y|V^{g}\right)+h\left(X|V^{g}\right)\\ \approx -\mu h\left(X|Y,U^{g}\right)+\left(\mu -1\right)h\left(X|U^{g},V^{g}\right)-h\left(Y|X,V^{g}\right)\\ \approx -\frac{\mu }{2}\log|D_{X|Y,U^{g}}|+\frac{\mu -1}{2}\log|D_{X|U^{g},V^{g}}|-\frac{1}{2}\log\left|D_{Y|X,V^{g}}\right|. \end{array}$$

Moreover,

(3) $$\begin{array}{c} D_{X|Y,U^{g}}={\left(\Sigma_{X}^{-1}+\Sigma_{Z}^{-1}+\Sigma_{1}^{-1}\right)}^{-1}, \end{array}$$

(4) $$\begin{array}{c} D_{Y|X,V^{g}}={\left(\Sigma_{Z}^{-1}+\Sigma_{2}^{-1}\right)}^{-1}, \end{array}$$

(5) $$\begin{array}{c} D_{X|U^{g},V^{g}}={\left(\Sigma_{X}^{-1}+\Sigma_{1}^{-1}+{\left(\Sigma_{Z}+\Sigma_{2}\right)}^{-1}\right)}^{-1}. \end{array}$$

Now, it is straightforward to write down the Gaussian version of the optimization problem in (2) with $\Sigma_{1}$ and $\Sigma_{2}$ as variables. However, as shown in the sequel, through a judicious change of variables, one can obtain an equivalent version that is more amenable to analysis.

Given λ∈(0,1), we introduce two random (column) vectors $M_{X}$ and $M_{Y}$, independent of $\left(X,Y,U^{g},V^{g}\right)$, such that the joint distribution of $\left(M_{X},M_{Y}\right)$ is the same as that of

$$\begin{array}{c} \left(\sqrt{\frac{1-\lambda }{\lambda }}\left(X-E\left[X|U^{g},V^{g}\right]\right),-\sqrt{\frac{\lambda }{1-\lambda }}\left(Y-E\left[Y|U^{g},V^{g}\right]\right)\right). \end{array}$$

Denote the covariance matrix of ${\left(M_{X}^{t},M_{Y}^{t}\right)}^{t}$ by $\Sigma_{{\left(M_{X}^{t},M_{Y}^{t}\right)}^{t}}$. We have

$$\begin{array}{cc} \Sigma_{{\left(M_{X}^{t},M_{Y}^{t}\right)}^{t}}^{-1} & =\left(\begin{array}{cc} \sqrt{\frac{\lambda }{1-\lambda }}I & 0\\ 0 & -\sqrt{\frac{1-\lambda }{\lambda }}I \end{array}\right)\left(\Sigma_{{\left(X^{t},Y^{t}\right)}^{t}}^{-1}+\Sigma_{{\left(N_{1}^{t},N_{2}^{t}\right)}^{t}}^{-1}\right)\left(\begin{array}{cc} \sqrt{\frac{\lambda }{1-\lambda }}I & 0\\ 0 & -\sqrt{\frac{1-\lambda }{\lambda }}I \end{array}\right)\\ & =\left(\begin{array}{cc} \frac{\lambda }{1-\lambda }\left(\Sigma_{X}^{-1}+\Sigma_{Z}^{-1}+\Sigma_{1}^{-1}\right) & \Sigma_{Z}^{-1}\\ \Sigma_{Z}^{-1} & \frac{1-\lambda }{\lambda }\left(\Sigma_{Z}^{-1}+\Sigma_{2}^{-1}\right) \end{array}\right). \end{array}$$

Define $W_{X}=X+M_{X}$ and $W_{Y}=Y+M_{Y}$. Since

(6) $$\begin{array}{cc} D_{{\left(X^{t},Y^{t}\right)}^{t}|W_{X},W_{Y}} & ={\left(\Sigma_{{\left(X^{t},Y^{t}\right)}^{t}}^{-1}+\Sigma_{M_{X},M_{Y}}^{-1}\right)}^{-1}\\ & ={\left(\begin{array}{cc} \Sigma_{X}^{-1}+\Sigma_{Z}^{-1}+\frac{\lambda }{1-\lambda }\left(\Sigma_{X}^{-1}+\Sigma_{Z}^{-1}+\Sigma_{1}^{-1}\right) & 0\\ 0 & \Sigma_{Z}^{-1}+\frac{1-\lambda }{\lambda }\left(\Sigma_{Z}^{-1}+\Sigma_{2}^{-1}\right) \end{array}\right)}^{-1}, \end{array}$$

X and Y must be conditionally independent given $\left(W_{X},W_{Y}\right)$. It can be verified that

$$\begin{array}{c} D_{X}^{g}:=D_{X|W_{X},W_{Y},U^{g}}={\left(D_{X|W_{X},W_{Y}}^{-1}+\Sigma_{1}^{-1}\right)}^{-1}=\left(1-\lambda \right){\left(\Sigma_{X}^{-1}+\Sigma_{Z}^{-1}+\Sigma_{1}^{-1}\right)}^{-1},\\ D_{Y}^{g}:=D_{Y|W_{X},W_{Y},V^{g}}={\left(D_{Y|W_{X},W_{Y}}^{-1}+\Sigma_{2}^{-1}\right)}^{-1}=\lambda {\left(\Sigma_{Z}^{-1}+\Sigma_{2}^{-1}\right)}^{-1}, \end{array}$$

which implies

(7) $$\begin{array}{c} \Sigma_{1}={\left(\left(1-\lambda \right)D_{X}^{-1}-\Sigma_{X}^{-1}-\Sigma_{Z}^{-1}\right)}^{-1}, \end{array}$$

(8) $$\begin{array}{c} \Sigma_{2}={\left(\lambda {\left(D_{Y}^{g}\right)}^{-1}-\Sigma_{Z}^{-1}\right)}^{-1}. \end{array}$$

Substituting (7) and (8) into (3–(5) gives

(9) $$\begin{array}{c} D_{X|Y,U^{g}}=\frac{1}{1-\lambda }D_{X}^{g}, \end{array}$$

(10) $$\begin{array}{c} D_{Y|X,V^{g}}=\frac{1}{\lambda }D_{Y}^{g}, \end{array}$$

(11) $$\begin{array}{c} D_{X|U^{g},V^{g}}=\frac{1}{1-\lambda }{\left({\left(D_{X}^{g}\right)}^{-1}-\frac{1}{\lambda (1-\lambda )}\Sigma_{Z}^{-1}D_{Y}^{g}\Sigma_{Z}^{-1}\right)}^{-1}. \end{array}$$

Therefore, the Gaussian version of the optimization problem in (2) can be written as

(12) $$\begin{array}{c} \underset{\left(D_{X}^{g},D_{Y}^{g}\right)\in D^{\prime }}{\inf}-\frac{\mu }{2}\log|D_{X}^{g}|-\frac{\mu -1}{2}\log\left|{\left(D_{X}^{g}\right)}^{-1}-\frac{1}{\lambda (1-\lambda )}\Sigma_{Z}^{-1}D_{Y}^{g}\Sigma_{Z}^{-1}\right|-\frac{1}{2}\log\left|D_{Y}^{g}\right|, \end{array}$$

where $D^{\prime }=\left\{\left(D_{a},D_{b}\right):0≺D_{a}⪯\left(1-\lambda \right)D_{1},0≺D_{b}⪯\lambda D_{2}\right\}$. It is clear that the infimum in (12) is achievable by some $\left(D_{X}^{*},D_{Y}^{*}\right)\in D^{\prime }$. Moreover, such $\left(D_{X}^{*},D_{Y}^{*}\right)$ must satisfy the following KKT conditions:

(13) $$\begin{array}{c} -\mu {\left(D_{X}^{*}\right)}^{-1}+\left(\mu -1\right){\left(D_{X}^{*}\right)}^{-1}{\left({\left(D_{X}^{*}\right)}^{-1}-\frac{1}{\lambda (1-\lambda )}\Sigma_{Z}^{-1}D_{Y}^{*}\Sigma_{Z}^{-1}\right)}^{-1}{\left(D_{X}^{*}\right)}^{-1}+\Pi_{1}=0, \end{array}$$

(14) $$\begin{array}{c} \frac{\left(\mu -1\right)}{\lambda (1-\lambda )}\Sigma_{Z}^{-1}{\left({\left(D_{X}^{*}\right)}^{-1}-\frac{1}{\lambda (1-\lambda )}\Sigma_{Z}^{-1}D_{Y}^{*}\Sigma_{Z}^{-1}\right)}^{-1}\Sigma_{Z}^{-1}-{\left(D_{Y}^{*}\right)}^{-1}+\Pi_{2}=0, \end{array}$$

(15) $$\begin{array}{c} \Pi_{1}\left(D_{X}^{*}-\left(1-\lambda \right)D_{1}\right)=0, \end{array}$$

(16) $$\begin{array}{c} \Pi_{2}\left(D_{Y}^{*}-\lambda D_{2}\right)=0, \end{array}$$

where $\Pi_{1},\Pi_{2}⪰0$.

## 3. The Key Construction

Let $\left(X^{*},Y^{*}\right)$ be an identically distributed copy of (X,Y). Moreover, let $N_{1}^{*}$ and $N_{2}^{*}$ be two *n*-dimensional zero-mean Gaussian random (column) vectors with covariance matrices $\Sigma_{1}^{*}$ and $\Sigma_{2}^{*}$, respectively. It is assumed that $\left(X^{*},Y^{*}\right)$, $N_{1}^{*}$, and $N_{2}^{*}$ are mutually independent. Define $U^{*}=X^{*}+N_{1}^{*}$ and $V^{*}=Y^{*}+N_{2}^{*}$. We choose $\Sigma_{1}^{*}$ and $\Sigma_{2}^{*}$ such that (cf. (9)–(11))

(17) $$\begin{array}{cc} D_{X^{*}|Y^{*},U^{*}} & =\frac{1}{1-\lambda }D_{X}^{*}, \end{array}$$

(18) $$\begin{array}{cc} D_{Y^{*}|X^{*},V^{*}} & =\frac{1}{\lambda }D_{Y}^{*}, \end{array}$$

(19) $$\begin{array}{cc} D_{X^{*}|U^{*},V^{*}} & =\frac{1}{1-\lambda }{\left({\left(D_{X}^{*}\right)}^{-1}-\frac{1}{\lambda (1-\lambda )}\Sigma_{Z}^{-1}D_{Y}^{*}\Sigma_{Z}^{-1}\right)}^{-1} \end{array}$$

for some $\left(D_{X}^{*},D_{Y}^{*}\right)\in D^{\prime }$ satisfying the KKT conditions (13)–(16).

Let *U* and *V* be two arbitrary random objects jointly distributed with (X,Y) such that U↔X↔Y↔V and $(D_{X|Y,U},D_{Y|X,V})\in D$. It is assumed that (X,Y,U,V) and $\left(X^{*},Y^{*},U^{*},V^{*}\right)$ are mutually independent. We aim to show that the objective function in (2) does not increase when (X,Y,U,V) is replaced with $\left(X^{*},Y^{*},U^{*},V^{*}\right)$, from which the desired result follows immediately. To this end, we develop a monotone path argument based on the following construction.

For λ∈[0,1], define

$$\begin{array}{c} \left(\begin{array}{c} X_{\lambda }\\ {\bar{X}}_{\lambda } \end{array}\right)=\left(\begin{array}{cc} \sqrt{\lambda }I & \sqrt{1-\lambda }I\\ \sqrt{1-\lambda }I & -\sqrt{\lambda }I \end{array}\right)\left(\begin{array}{c} X\\ X^{*} \end{array}\right),\\ \left(\begin{array}{c} Y_{\lambda }\\ {\bar{Y}}_{\lambda } \end{array}\right)=\left(\begin{array}{cc} \sqrt{\lambda }I & \sqrt{1-\lambda }I\\ \sqrt{1-\lambda }I & -\sqrt{\lambda }I \end{array}\right)\left(\begin{array}{c} Y\\ Y^{*} \end{array}\right). \end{array}$$

**Figure 1:**
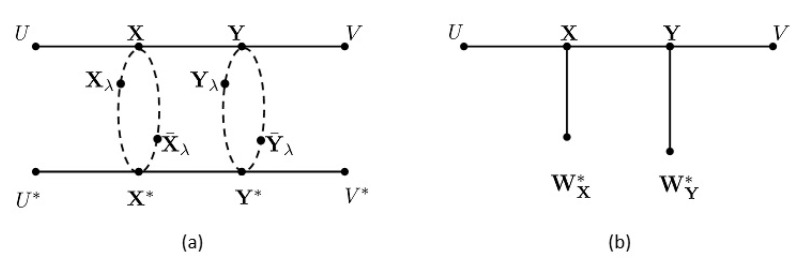
Illustrations of the Markov structures in (20) and (21).

It is easy to verify the following Markov structures (see Figure 1a):

(20) $$\begin{array}{c} \left\{\begin{array}{c} \left(U,U^{*}\right)↔\left(X,X^{*}\right)↔\left(X_{\lambda },{\bar{Y}}_{\lambda }\right)↔\left(Y,Y^{*}\right)↔\left(V,V^{*}\right),\\ \left(U,U^{*}\right)↔\left(X,X^{*}\right)↔\left({\bar{X}}_{\lambda },Y_{\lambda }\right)↔\left(Y,Y^{*}\right)↔\left(V,V^{*}\right), \end{array}\right.\;\lambda \in \left[0,1\right]. \end{array}$$

Note that, as λ changes from 0 to 1, $X_{\lambda }$ ($Y_{\lambda }$) moves from $X^{*}$ ($Y^{*}$) to X (Y) while ${\bar{X}}_{\lambda }$ (${\bar{Y}}_{\lambda }$) moves the other way around. This construction generalizes its counterpart in the doubling trick, which corresponds to the special case $\lambda =\frac{1}{2}$.

For λ∈(0,1), define $W_{X}^{*}=X+M_{X}^{*}$ and $W_{Y}^{*}=Y+M_{Y}^{*}$, where

$$\begin{array}{c} M_{X}^{*}=\sqrt{\frac{1-\lambda }{\lambda }}\left(X^{*}-E\left[X^{*}|U^{*},V^{*}\right]\right),\\ M_{Y}^{*}=-\sqrt{\frac{\lambda }{1-\lambda }}\left(Y^{*}-E\left[Y^{*}|U^{*},V^{*}\right]\right). \end{array}$$

In view of (6), we have the following Markov structure (see Figure 1b):

(21) $$\begin{array}{c} U↔X↔(W_{X}^{*},W_{Y}^{*})↔Y↔V. \end{array}$$

Define $D_{X}=D_{X|X_{\lambda },{\bar{Y}}_{\lambda },U,U^{*}}$ and $D_{Y}=D_{Y|X_{\lambda },{\bar{Y}}_{\lambda },V,V^{*}}$. It can be verified that for λ∈(0,1),

(22) $$\begin{array}{cc} D_{X} & =D_{X|X_{\lambda },{\bar{Y}}_{\lambda },U,U^{*},V,V^{*}} \end{array}$$

(23) $$\begin{array}{c} =D_{X|W_{X}^{*},W_{Y}^{*},U,U^{*},V,V^{*}} \end{array}$$

(24) $$\begin{array}{c} =D_{X|W_{X}^{*},W_{Y}^{*},U,V} \end{array}$$

(25) $$\begin{array}{c} =D_{X|W_{X}^{*},W_{Y}^{*},U}, \end{array}$$

where (22) is due to (20), (23) is due to the existence of a bijection between $\left(X_{\lambda },{\bar{Y}}_{\lambda },U^{*},V^{*}\right)$ and $\left(W_{X}^{*},W_{Y}^{*},U^{*},V^{*}\right)$, (24) is due to the fact that $\left(U^{*},V^{*}\right)$ is independent of $\left(X,W_{X},W_{Y},U,V\right)$, and (25) is due to (21). Similarly, we have $D_{Y}=D_{Y|W_{X}^{*},W_{Y}^{*},V}$ for λ∈(0,1).

## 4. Proof of Theorem 1

The following technical lemmas are needed for proving Theorem 1. Their proofs are relegated to the Appendix A, Appendix B, Appendix C and Appendix D.

**Lemma** **1.** *For λ∈(0,1),*

(26) $$\begin{array}{c} \frac{d}{d\lambda }h\left(X_{\lambda }|U,U^{*},V,V^{*}\right)=-\frac{n}{2(1-\lambda )}+\frac{1}{2{\left(1-\lambda \right)}^{2}}tr\left(D_{X^{*}|U^{*},V^{*}}^{-1}D_{X|X_{\lambda },U,U^{*},V,V^{*}}\right), \end{array}$$

(27) $$\begin{array}{c} \frac{d}{d\lambda }h\left(X_{\lambda },{\bar{Y}}_{\lambda }|U,U^{*}\right)=-\frac{n}{2(1-\lambda )}+\frac{1}{2{\left(1-\lambda \right)}^{2}}tr\left(D_{X^{*}|Y^{*},U^{*}}^{-1}D_{X}\right), \end{array}$$

(28) $$\begin{array}{c} \frac{d}{d\lambda }h\left(X_{\lambda },{\bar{Y}}_{\lambda }|V,V^{*}\right)=\frac{n}{2\lambda }-\frac{1}{2\lambda^{2}}tr\left(D_{Y^{*}|X^{*},V^{*}}^{-1}D_{Y}\right). \end{array}$$

**Lemma** **2.** *For λ∈(0,1),*

$$\begin{array}{c} D_{X|X_{\lambda },U,U^{*},V,V^{*}}⪰\Delta ≻0, \end{array}$$

*where*

$$\begin{array}{c} \Delta ={\left(D_{X}^{-1}-\frac{1}{{\left(1-\lambda \right)}^{2}}\Sigma_{Z}^{-1}D_{Y}^{*}{\left(\frac{1}{1-\lambda }D_{Y}^{*}-D_{Y}\right)}^{-1}D_{Y}^{*}\Sigma_{Z}^{-1}\right)}^{-1}. \end{array}$$

**Lemma** **3.** *${\left(B^{-1}-A^{-1}\right)}^{-1}$ is matrix convex in (A,B) for A≻B≻0.*

**Lemma** **4.** *Let X be a Gaussian random vector and U be an arbitrary random object. Moreover, let $N_{1}$ and $N_{2}$ be two zero-mean Gaussian random vectors, independent of (X,U), with covariance matrices $\Sigma_{1}$ and $\Sigma_{2}$ respectively. If $\Sigma_{2}≻\Sigma_{1}≻0$, then*

$$\begin{array}{c} D_{X|X+N_{2},U}⪰{\left(D_{X|X+N_{1},U}^{-1}+\Sigma_{2}^{-1}-\Sigma_{1}^{-1}\right)}^{-1}. \end{array}$$

Now, we are in a position to prove Theorem 1. Define

$$\begin{array}{cc} h_{\lambda }= & -\mu h\left({\bar{X}}_{\lambda }|X_{\lambda },U,U^{*}\right)+\mu h\left({\bar{Y}}_{\lambda }|X_{\lambda },U,U^{*}\right)+\left(\mu -1\right)h\left({\bar{X}}_{\lambda }|X_{\lambda },U,U^{*},V,V^{*}\right)\\ & -h\left({\bar{Y}}_{\lambda }|X_{\lambda },V,V^{*}\right)+h\left({\bar{X}}_{\lambda }|X_{\lambda },V,V^{*}\right). \end{array}$$

Clearly,

$$\begin{array}{c} {\left.h_{\lambda }\right|}_{\lambda =0}=-\mu h\left(X|U\right)+\mu h\left(Y|U\right)+\left(\mu -1\right)h\left(X|U,V\right)-h\left(Y|V\right)+h\left(X|V\right),\\ {\left.h_{\lambda }\right|}_{\lambda =1}=-\mu h\left(X^{*}|U^{*}\right)+\mu h\left(Y^{*}|U^{*}\right)+\left(\mu -1\right)h\left(X^{*}|U^{*},V^{*}\right)-h\left(Y^{*}|V^{*}\right)+h\left(X^{*}|V^{*}\right). \end{array}$$

Therefore, it suffices to show $\frac{dh_{\lambda }}{d\lambda }\leq 0$ for λ∈(0,1). Note that

$$\begin{array}{cc} h_{\lambda }= & -\mu h\left(X_{\lambda },{\bar{X}}_{\lambda }|U,U^{*}\right)+\mu h\left(X_{\lambda },{\bar{Y}}_{\lambda }|U,U^{*}\right)+\left(\mu -1\right)h\left(X_{\lambda },{\bar{X}}_{\lambda }|U,U^{*},V,V^{*}\right)\\ & -\left(\mu -1\right)h\left(X_{\lambda }|U,U^{*},V,V^{*}\right)-h\left(X_{\lambda },{\bar{Y}}_{\lambda }|V,V^{*}\right)+h\left(X_{\lambda },{\bar{X}}_{\lambda }|V,V^{*}\right). \end{array}$$

Since $h\left(X_{\lambda },{\bar{X}}_{\lambda }|U,U^{*}\right)=h\left(X,X^{*}|U,U^{*}\right)$, $h\left(X_{\lambda },{\bar{X}}_{\lambda }|U,U^{*},V,V^{*}\right)=h\left(X,X^{*}|U,U^{*},V,V^{*}\right)$, and $h\left(X_{\lambda },{\bar{X}}_{\lambda }|V,V^{*}\right)=h\left(X,X^{*}|V,V^{*}\right)$, we have

$$\begin{array}{c} \frac{d}{d\lambda }h\left(X_{\lambda },{\bar{X}}_{\lambda }|U,U^{*}\right)=0,\\ \frac{d}{d\lambda }h\left(X_{\lambda },{\bar{X}}_{\lambda }|U,U^{*},V,V^{*}\right)=0,\\ \frac{d}{d\lambda }h\left(X_{\lambda },{\bar{X}}_{\lambda }|V,V^{*}\right)=0, \end{array}$$

which, together with Lemma 1, implies

(29) $$\begin{array}{cc} \frac{dh_{\lambda }}{d\lambda }= & -\frac{n}{2\lambda (1-\lambda )}+\frac{\mu }{2{\left(1-\lambda \right)}^{2}}\mathrm{tr}\left(D_{X^{*}|Y^{*},U^{*}}^{-1}D_{X}\right)+\frac{1}{2\lambda^{2}}\mathrm{tr}\left(D_{Y^{*}|X^{*},V^{*}}^{-1}D_{Y}\right)\\ & -\frac{\left(\mu -1\right)}{2{\left(1-\lambda \right)}^{2}}\mathrm{tr}\left(D_{X^{*}|U^{*},V^{*}}^{-1}D_{X|X_{\lambda },U,U^{*},V,V^{*}}\right). \end{array}$$

Combining Lemma 2 and (29) shows

(30) $$\begin{array}{c} \frac{dh_{\lambda }}{d\lambda }\leq -\frac{n}{2\lambda (1-\lambda )}-\frac{1}{2}f\left(D_{X},D_{Y}\right), \end{array}$$

where

$$\begin{array}{c} f\left(D_{X},D_{Y}\right)=-\frac{\mu }{{\left(1-\lambda \right)}^{2}}\mathrm{tr}\left(D_{X^{*}|Y^{*},U^{*}}^{-1}D_{X}\right)-\frac{1}{\lambda^{2}}\mathrm{tr}\left(D_{Y^{*}|X^{*},V^{*}}^{-1}D_{Y}\right)+\frac{\left(\mu -1\right)}{{\left(1-\lambda \right)}^{2}}\mathrm{tr}\left(D_{X^{*}|U^{*},V^{*}}^{-1}\Delta \right). \end{array}$$

We shall derive a lower bound for $f(D_{X},D_{Y})$. To this end, we first identify certain constraints on $D_{X}$ and $D_{Y}$. Let $W=X+\tilde{Z}$, where $\tilde{Z}$ is a zero-mean Gaussian random vector, independent of (X,U), with covariance matrix $\Sigma_{\tilde{Z}}$. We shall choose $\Sigma_{\tilde{Z}}$ such that $D_{X|W}=D_{X|W_{X}^{*},W_{Y}^{*}}$, which implies

(31) $$\begin{array}{c} \Sigma_{\tilde{Z}}≺\Sigma_{Z}, \end{array}$$

(32) $$\begin{array}{c} D_{X|W,U}=D_{X|W_{X}^{*},W_{Y}^{*},U}. \end{array}$$

It can be verified (cf. (6)) that

(33) $$\begin{array}{cc} \Sigma_{\tilde{Z}} & ={\left(\Sigma_{Z}^{-1}+\frac{\lambda }{1-\lambda }\left(\Sigma_{X}^{-1}+\Sigma_{Z}^{-1}+{\left(\Sigma_{1}^{*}\right)}^{-1}\right)\right)}^{-1}\\ & ={\left(\Sigma_{Z}^{-1}+\lambda {\left(D_{X}^{*}\right)}^{-1}\right)}^{-1}. \end{array}$$

In view of (25), (31), (32), and Lemma 4,

$$\begin{array}{c} D_{X|Y,U}⪰{\left(D_{X}^{-1}+\Sigma_{Z}^{-1}-\Sigma_{\tilde{Z}}^{-1}\right)}^{-1}, \end{array}$$

which, together with (33) and the constraint $0≺D_{X|Y,U}⪯D_{1}$, implies

$$\begin{array}{c} D_{X}⪯{\left(D_{1}^{-1}+\lambda {\left(D_{X}^{*}\right)}^{-1}\right)}^{-1}. \end{array}$$

Similarly, we have

(34) $$\begin{array}{c} D_{Y}⪯{\left(D_{2}^{-1}+\left(1-\lambda \right)D_{Y}^{*-1}\right)}^{-1}. \end{array}$$

Define ${\bar{D}}_{1}={\left(D_{1}^{-1}+\lambda {\left(D_{X}^{*}\right)}^{-1}\right)}^{-1}$, ${\bar{D}}_{2}={\left(D_{2}^{-1}+\left(1-\lambda \right)D_{Y}^{*-1}\right)}^{-1}$, and $\bar{D}=\left\{\left(D_{a},D_{b}\right):0≺D_{a}⪯{\bar{D}}_{1},0≺D_{b}⪯{\bar{D}}_{2}\right\}$. Consider

$$\begin{array}{c} \min_{\left(D_{X},D_{Y}\right)\in \bar{D}}f\left(D_{X},D_{Y}\right), \end{array}$$

which is a convex semidefinite programming problem according to Lemma 3 (in view of (34) and Lemma 2, we have $D_{Y}≺\frac{1}{1-\lambda }D_{Y}^{*}$ and Δ≻0). Note that $\left(D_{X},D_{Y}\right)\in \bar{D}$ is an optimal solution to this convex programming problem if and only if it satisfies the following KKT conditions:

(35) $$\begin{array}{c} -\frac{\mu }{{\left(1-\lambda \right)}^{2}}D_{X^{*}|Y^{*},U^{*}}^{-1}+\frac{\left(\mu -1\right)}{{\left(1-\lambda \right)}^{2}}\left(D_{X}^{-1}\Delta D_{X^{*}|U^{*},V^{*}}^{-1}\Delta D_{X}^{-1}\right)+\Pi_{1}^{\prime }=0\\ -\frac{1}{\lambda^{2}}D_{Y^{*}|X^{*},V^{*}}^{-1}+\frac{\left(\mu -1\right)}{{\left(1-\lambda \right)}^{2}}\left({\left(D_{Y}^{*}-\left(1-\lambda \right)D_{Y}\right)}^{-1}D_{Y}^{*}\Sigma_{Z}^{-1}\Delta D_{X^{*}|U^{*},V^{*}}^{-1}\Delta \Sigma_{Z}^{-1}D_{Y}^{*}{\left(D_{Y}^{*}-\left(1-\lambda \right)D_{Y}\right)}^{-1}\right) \end{array}$$

(36) $$\begin{array}{c} \;+\Pi_{2}^{\prime }=0, \end{array}$$

(37) $$\begin{array}{c} \;\Pi_{1}^{\prime }\left(D_{X}-{\bar{D}}_{1}\right)=0, \end{array}$$

(38) $$\begin{array}{c} \;\Pi_{2}^{\prime }\left(D_{Y}-{\bar{D}}_{2}\right)=0, \end{array}$$

where $\Pi_{1}^{\prime },\Pi_{2}^{\prime }⪰0$. Let $D_{X}=D_{X}^{*}$, $D_{Y}=D_{Y}^{*}$, $\Pi_{1}^{\prime }=\frac{1}{1-\lambda }\Pi_{1}$, and $\Pi_{2}^{\prime }=\frac{1}{\lambda }\Pi_{2}$. One can readily verify by leveraging (17)–(19) that (35)–(38) with this particular choice of $\left(D_{X},D_{Y},\Pi_{1}^{\prime },\Pi_{2}^{\prime }\right)$ can be deduced from (13)–(16); it is also easy to see that $\left(D_{X}^{*},D_{Y}^{*}\right)\in \bar{D}$. Therefore,

(39) $$\begin{array}{c} \min_{\left(D_{X},D_{Y}\right)\in \bar{D}}f\left(D_{X},D_{Y}\right)=f\left(D_{X}^{*},D_{Y}^{*}\right). \end{array}$$

Combining (30), (39), and the fact that $f\left(D_{X}^{*},D_{Y}^{*}\right)=-\frac{n}{\lambda (1-\lambda )}$ shows $\frac{dh_{\lambda }}{d\lambda }\leq 0$, which completes the proof.

## 5. Conclusions

We have generalized an extremal result by Courtade and Jiao. It is worth mentioning that recently Courtade [24] found a different generalization using the doubling trick. So far, we have not been able to prove this new result via the monotone path argument. A deeper understanding of the connection between these two methods is needed. It is conceivable that the convex-like property revealed by the monotone path argument and the subadditive property revealed by the doubling trick are manifestations of a common underlying mathematical structure yet to be uncovered.

## Appendix A. Proof of Lemma 1

Note that

(A1) $$\begin{array}{cc} \frac{d}{d\lambda }h\left(X_{\lambda }|U,U^{*},V,V^{*}\right) & =\frac{d}{d\lambda }\left(\frac{n}{2}\log\lambda +\frac{d}{d\lambda }h\left(X+M_{X}^{*}|U,V\right)\right)\\ & =\frac{n}{2\lambda }+\frac{d}{d\lambda }h\left(X+M_{X}^{*}|U,V\right)\\ & =\frac{n}{2\lambda }+tr\left(\nabla_{\Sigma_{M_{X}^{*}}}h\left(X+M_{X}^{*}|U,V\right)\nabla_{\lambda }\Sigma_{M_{X}^{*}}\right)\\ & =\frac{n}{2\lambda }-\frac{1}{\lambda^{2}}tr\left(D_{X^{*}|U^{*},V^{*}}\nabla_{\Sigma_{M_{X}^{*}}}h\left(X+M_{X}^{*}|U,V\right)\right), \end{array}$$

where $\Sigma_{M_{X}^{*}}=\frac{1-\lambda }{\lambda }D_{X^{*}|U^{*},V^{*}}$. We have

(A2) $$\begin{array}{cc} \nabla_{\Sigma_{M_{X}^{*}}}h\left(X+M_{X}^{*}|U,V\right) & =\frac{1}{2}J\left(X+M_{X}^{*}|U,V\right) \end{array}$$

(A3) $$\begin{array}{c} =\frac{\lambda }{2}J\left(X_{\lambda }|U,U^{*},V,V^{*}\right), \end{array}$$

where J(·) denotes the Fisher information matrix, and (A2) is due to ([25], Theorem 4). Substituting (A3) into (A1) gives

(A4) $$\begin{array}{c} \frac{d}{d\lambda }h\left(X_{\lambda }|U,U^{*},V,V^{*}\right)=\frac{n}{2\lambda }-\frac{1}{2\lambda }\mathrm{tr}\left(D_{X^{*}|U^{*},V^{*}}J\left(X_{\lambda }|U,U^{*},V,V^{*}\right)\right), \end{array}$$

which, together with the fact ([26], Lemma 2) that

(A5) $$\begin{array}{c} J\left(X_{\lambda }|U,U^{*},V,V^{*}\right)=\frac{1}{1-\lambda }D_{X^{*}|U^{*},V^{*}}^{-1}-\frac{\lambda }{{\left(1-\lambda \right)}^{2}}D_{X^{*}|U^{*},V^{*}}^{-1}D_{X|X_{\lambda },U,U^{*},V,V^{*}}D_{X^{*}|U^{*},V^{*}}^{-1}, \end{array}$$

proves (26).

It remains to prove (27) since (28) follows by symmetry. Note that

$$\begin{array}{c} \left(\begin{array}{c} X_{\lambda }\\ {\bar{Y}}_{\lambda } \end{array}\right)=\left(\begin{array}{cc} \sqrt{\lambda }I & 0\\ 0 & \sqrt{1-\lambda }I \end{array}\right)\left(\left(\begin{array}{c} X\\ Y \end{array}\right)+\left(\begin{array}{c} N_{X}\\ N_{Y} \end{array}\right)\right), \end{array}$$

where $N_{X}=\sqrt{\frac{1-\lambda }{\lambda }}X^{*}$ and $N_{Y}=-\sqrt{\frac{\lambda }{1-\lambda }}Y^{*}$. Define

$$\begin{array}{c} {\bar{N}}_{X}=N_{X}-E\left[N_{X}|U^{*}\right],\\ {\bar{N}}_{Y}=N_{Y}-E\left[N_{Y}|U^{*}\right]. \end{array}$$

Denote the covariance matrix of ${\left({\bar{N}}_{X}^{t},{\bar{N}}_{Y}^{t}\right)}^{t}$ by $\Sigma_{{\left({\bar{N}}_{X}^{t},{\bar{N}}_{Y}^{t}\right)}^{t}}$. It can be verified (cf. (A4) and (A5)) that

(A6) $$\begin{array}{c} \frac{d}{d\lambda }h\left(X_{\lambda },{\bar{Y}}_{\lambda }|U,U^{*}\right)=\frac{(1-2\lambda )n}{2\lambda (1-\lambda )}+\frac{1}{2}\mathrm{tr}\left(\nabla_{\lambda }\Sigma_{{\left({\bar{N}}_{X}^{t},{\bar{N}}_{Y}^{t}\right)}^{t}}J\left({\left(X^{t},Y^{t}\right)}^{t}+{\left({\bar{N}}_{X}^{t},{\bar{N}}_{Y}^{t}\right)}^{t}|U\right)\right), \end{array}$$

(A7) $$\begin{array}{c} J\left({\left(X^{t},Y^{t}\right)}^{t}+{\left({\bar{N}}_{X}^{t},{\bar{N}}_{Y}^{t}\right)}^{t}|U\right)=\Sigma_{{\left({\bar{N}}_{X}^{t},{\bar{N}}_{Y}^{t}\right)}^{t}}^{-1}-\Sigma_{{\left({\bar{N}}_{X}^{t},{\bar{N}}_{Y}^{t}\right)}^{t}}^{-1}D_{{\left(X^{t},Y^{t}\right)}^{t}|X_{\lambda },{\bar{Y}}_{\lambda },U,U^{*}}\Sigma_{{\left({\bar{N}}_{X}^{t},{\bar{N}}_{Y}^{t}\right)}^{t}}^{-1}. \end{array}$$

Since

$$\begin{array}{c} \Sigma_{{\left({\bar{N}}_{X}^{t},{\bar{N}}_{Y}^{t}\right)}^{t}}=\left(\begin{array}{cc} \frac{1-\lambda }{\lambda }D_{X^{*}|U^{*}} & -D_{X^{*}|U^{*}}\\ -D_{X^{*}|U^{*}} & \frac{\lambda }{1-\lambda }\left(D_{X^{*}|U^{*}}+\Sigma_{Z}\right) \end{array}\right), \end{array}$$

we have

$$\begin{array}{c} \Sigma_{{\left({\bar{N}}_{X}^{t},{\bar{N}}_{Y}^{t}\right)}^{t}}^{-1}=\left(\begin{array}{cc} \frac{\lambda }{1-\lambda }\left(D_{X^{*}|U^{*}}^{-1}+\Sigma_{Z}^{-1}\right) & \Sigma_{Z}^{-1}\\ \Sigma_{Z}^{-1} & \frac{1-\lambda }{\lambda }\Sigma_{Z}^{-1} \end{array}\right),\\ \nabla_{\lambda }\Sigma_{{\left({\bar{N}}_{X}^{t},{\bar{N}}_{Y}^{t}\right)}^{t}}=\left(\begin{array}{cc} -\frac{1}{\lambda^{2}}D_{X^{*}|U^{*}} & 0\\ 0 & \frac{1}{{\left(1-\lambda \right)}^{2}}\left(D_{X^{*}|U^{*}}+\Sigma_{Z}\right) \end{array}\right), \end{array}$$

which further implies

(A8) $$\begin{array}{c} \frac{1}{2}\mathrm{tr}\left(\nabla_{\lambda }\Sigma_{{\left({\bar{N}}_{X}^{t},{\bar{N}}_{Y}^{t}\right)}^{t}}\Sigma_{{\left({\bar{N}}_{X}^{t},{\bar{N}}_{Y}^{t}\right)}^{t}}^{-1}\right)=0. \end{array}$$

Combining (A6)–(A8) gives

(A9) $$\begin{array}{c} \frac{d}{d\lambda }h\left(X_{\lambda },{\bar{Y}}_{\lambda }|U,U^{*}\right)=\frac{(1-2\lambda )n}{2\lambda (1-\lambda )}-\frac{1}{2}\mathrm{tr}\left(\Sigma_{{\left({\bar{N}}_{X}^{t},{\bar{N}}_{Y}^{t}\right)}^{t}}^{-1}\nabla_{\lambda }\Sigma_{{\left({\bar{N}}_{X}^{t},{\bar{N}}_{Y}^{t}\right)}^{t}}\Sigma_{{\left({\bar{N}}_{X}^{t},{\bar{N}}_{Y}^{t}\right)}^{t}}^{-1}D_{{\left(X^{t},Y^{t}\right)}^{t}|X_{\lambda },{\bar{Y}}_{\lambda },U,U^{*}}\right). \end{array}$$

In view of (20),

(A10) $$\begin{array}{c} D_{{\left(X^{t},Y^{t}\right)}^{t}|X_{\lambda },{\bar{Y}}_{\lambda },U,U^{*}}=\left(\begin{array}{cc} D_{X} & 0\\ 0 & D_{Y|X_{\lambda },{\bar{Y}}_{\lambda }} \end{array}\right). \end{array}$$

Moreover,

(A11) $$\begin{array}{cc} \Sigma_{{\left({\bar{N}}_{X}^{t},{\bar{N}}_{Y}^{t}\right)}^{t}}^{-1}\nabla_{\lambda }\Sigma_{{\left({\bar{N}}_{X}^{t},{\bar{N}}_{Y}^{t}\right)}^{t}}\Sigma_{{\left({\bar{N}}_{X}^{t},{\bar{N}}_{Y}^{t}\right)}^{t}}^{-1} & =\left(\begin{array}{cc} \frac{1}{{\left(1-\lambda \right)}^{2}}\left(D_{X^{*}|U^{*}}^{-1}+\Sigma_{Z}^{-1}\right) & 0\\ 0 & \frac{1}{\lambda^{2}}\Sigma_{Z}^{-1} \end{array}\right)\\ & =\left(\begin{array}{cc} \frac{1}{{\left(1-\lambda \right)}^{2}}D_{X^{*}|Y^{*},U^{*}}^{-1} & 0\\ 0 & \frac{1}{\lambda^{2}}\Sigma_{Z}^{-1} \end{array}\right). \end{array}$$

Substituting (A10) and (A11) into (A9) yields

(A12) $$\begin{array}{c} \frac{d}{d\lambda }h\left(X_{\lambda },{\bar{Y}}_{\lambda }|U,U^{*}\right)=\frac{(1-2\lambda )n}{2\lambda (1-\lambda )}+\frac{1}{2{\left(1-\lambda \right)}^{2}}\mathrm{tr}\left(D_{X^{*}|Y^{*},U^{*}}^{-1}D_{X}\right)-\frac{1}{2\lambda^{2}}\mathrm{tr}\left(\Sigma_{Z}^{-1}D_{Y|X_{\lambda },{\bar{Y}}_{\lambda }}\right). \end{array}$$

It can be verified that

$$\begin{array}{cc} D_{{\left(X^{t},Y^{t}\right)}^{t}|X_{\lambda },{\bar{Y}}_{\lambda }} & =D_{{\left(X^{t},Y^{t}\right)}^{t}|X+N_{X},Y+N_{Y}}\\ & ={\left(\Sigma_{{\left(X^{t},Y^{t}\right)}^{t}}^{-1}+\Sigma_{{\left(N_{X}^{t},N_{Y}^{t}\right)}^{t}}^{-1}\right)}^{-1}\\ & =\left(\begin{array}{cc} \left(1-\lambda \right){\left(\Sigma_{X}^{-1}+\Sigma_{Z}^{-1}\right)}^{-1} & 0\\ 0 & \lambda \Sigma_{Z} \end{array}\right), \end{array}$$

which implies

(A13) $$\begin{array}{c} D_{Y|X_{\lambda },{\bar{Y}}_{\lambda }}=\lambda \Sigma_{Z}. \end{array}$$

Substituting (A13) into (A12) proves (27).

## Appendix B. Proof of Lemma 2

It can be verified that $E\left[W_{Y}^{*}|X,Y,W_{X}^{*}\right]=AX+Y-AW_{X}^{*}$, where $A=\frac{1}{1-\lambda }D_{Y}^{*}\Sigma_{Z}^{-1}$; moreover, $Q:=W_{Y}^{*}-E\left[W_{Y}^{*}|X,Y,W_{X}^{*}\right]$ is a zero-mean Gaussian random vector with covariance matrix $\Sigma_{Q}=\frac{1}{1-\lambda }D_{Y}^{*}$, and is independent of $\left(X,Y,W_{X}^{*},U,V\right)$. Define $\tilde{Y}=AX+Y$. We have

(A14) $$\begin{array}{cc} D_{{\left(X^{t},{\tilde{Y}}^{t}\right)}^{t}|W_{X}^{*},W_{Y}^{*},U,V} & =\left(\begin{array}{cc} I & 0\\ A & I \end{array}\right)D_{{\left(X^{t},Y^{t}\right)}^{t}|W_{X}^{*},W_{Y}^{*},U,V}\left(\begin{array}{cc} I & A^{t}\\ 0 & I \end{array}\right)\\ & =\left(\begin{array}{cc} I & 0\\ A & I \end{array}\right)\left(\begin{array}{cc} D_{X} & 0\\ 0 & D_{Y} \end{array}\right)\left(\begin{array}{cc} I & A^{t}\\ 0 & I \end{array}\right). \end{array}$$

On the other hand,

(A15) $$\begin{array}{cc} D_{{\left(X^{t},{\tilde{Y}}^{t}\right)}^{t}|W_{X}^{*},W_{Y}^{*},U,V} & =D_{{\left(X^{t},{\tilde{Y}}^{t}\right)}^{t}|W_{X}^{*},U,V,\tilde{Y}+Q}\\ & ⪯{\left(D_{{\left(X^{t},{\tilde{Y}}^{t}\right)}^{t}|W_{X}^{*},U,V}^{-1}+\left(\begin{array}{cc} 0 & 0\\ 0 & \Sigma_{Q}^{-1} \end{array}\right)\right)}^{-1}, \end{array}$$

where (A15) is due to the fact that the distortion covariance matrix incurred by the MMSE estimator of ${\left(X^{t},{\tilde{Y}}^{t}\right)}^{t}$ from $\left(W_{X}^{*},U,V,\tilde{Y}+Q\right)$ is no greater than (in the semidefinite sense) that incurred by the linear MMSE estimator of ${\left(X^{t},{\tilde{Y}}^{t}\right)}^{t}$ from $\left(E\left[{\left(X^{t},{\tilde{Y}}^{t}\right)}^{t}|W_{X}^{*},U,V\right],\tilde{Y}+Q\right)$. Combining (A14) and (A15) yields

$$\begin{array}{c} D_{{\left(X^{t},{\tilde{Y}}^{t}\right)}^{t}|W_{X}^{*},U,V}⪰{\left(\begin{array}{cc} D_{X}^{-1}+A^{t}D_{Y}^{-1}A & -A^{t}D_{Y}^{-1}\\ -D_{Y}^{-1}A & D_{Y}^{-1}-\Sigma_{Q}^{-1} \end{array}\right)}^{-1}. \end{array}$$

Comparing the upper-left submatrices on the two sides of the above inequality gives $D_{X|W_{X}^{*},U,V}⪰\Delta$, which, together with the fact that $D_{X|X_{\lambda },U,U^{*},V,V^{*}}=D_{X|W_{X}^{*},U,V}$, proves $D_{X|X_{\lambda },U,U^{*},V,V^{*}}⪰\Delta$. Moreover, we have

$$\begin{array}{c} 0≺D_{{\left(X^{t},Y^{\prime t}\right)}^{t}|\left(W_{X},U,V\right)}^{-1}⪯\left(\begin{array}{cc} D_{X}^{-1}+A^{t}D_{Y}^{-1}A & -A^{t}D_{Y}^{-1}\\ -D_{Y}^{-1}A & D_{Y}^{-1}-\Sigma_{Q}^{-1} \end{array}\right), \end{array}$$

where the second inequality is due to (A14) and (A15). Therefore, Δ, which is equal to the inverse of the upper-left submatrix of

$$\begin{array}{c} {\left(\begin{array}{cc} D_{X}^{-1}+A^{t}D_{Y}^{-1}A & -A^{t}D_{Y}^{-1}\\ -D_{Y}^{-1}A & D_{Y}^{-1}-\Sigma_{Q}^{-1} \end{array}\right)}^{-1}, \end{array}$$

must be positive definite.

## Appendix C. Proof of Lemma 3

It is known [27] that $BS^{-1}B$ is matrix convex in (S,B) for symmetric matrix B and positive definite matrix S. The desired conclusion follows from the fact that ${\left(B^{-1}-A^{-1}\right)}^{-1}=B+B{\left(A-B\right)}^{-1}B$ and that affine transformations preserve convexity.

## Appendix D. Proof of Lemma 4

Without loss of generality, we assume that $N_{2}=N_{1}+\tilde{N}$, where $\tilde{N}$ is a zero-mean Gaussian random vector with covariance matrix $\tilde{\Sigma }=\Sigma_{2}-\Sigma_{1}$ and is independent of $\left(X,N_{1},U\right)$. As a consequence, $E\left[N_{1}|N_{2}\right]=HN_{2}$, where $H={\left(\Sigma_{1}^{-1}+{\tilde{\Sigma }}^{-1}\right)}^{-1}{\tilde{\Sigma }}^{-1}$; moreover, $M:=N_{1}-E\left[N_{1}|N_{2}\right]$ is a zero-mean Gaussian random vector with covariance matrix $\Sigma_{M}={\left(\Sigma_{1}^{-1}+{\tilde{\Sigma }}^{-1}\right)}^{-1}$, and is independent of $\left(X,N_{2},U\right)$. Note that

(A16) $$\begin{array}{cc} J(X+N_{1}|X+N_{2},U) & =J\left(\left(I-H\right)X+M|X+N_{2},U\right)\\ & =\left(\Sigma_{1}^{-1}+{\tilde{\Sigma }}^{-1}\right)-\Sigma_{1}^{-1}D_{X|X+N_{1},U}\Sigma_{1}^{-1}, \end{array}$$

where the second equality follows by ([26], Lemma 2). On the other hand,

(A17) $$\begin{array}{cc} J(X+N_{1}|X+N_{2},U) & ⪰D_{X+N_{1}|X+N_{2},U}^{-1} \end{array}$$

$$\begin{array}{cc} & =D_{\left(I-H\right)X+M|X+N_{2},U}^{-1} \end{array}$$

(A18) $$\begin{array}{c} ={\left(\left(I-H\right)D_{X|X+N_{2},U}\left(I-H^{t}\right)+\Sigma_{M}\right)}^{-1}, \end{array}$$

where (A17) is due to the conditional version of the Cramer–Rao inequality. Combining (A16) and (A18) yields the desired result.
